# Salmonella Bacterial Monotherapy Reduces Autochthonous Prostate Tumor Burden in the TRAMP Mouse Model

**DOI:** 10.1371/journal.pone.0160926

**Published:** 2016-08-09

**Authors:** Robert A. Kazmierczak, Bettina Gentry, Tyler Mumm, Heide Schatten, Abraham Eisenstark

**Affiliations:** 1 Cancer Research Center, Columbia, Missouri, United States of America; 2 Division of Biological Sciences, University of Missouri, Columbia, Missouri, United States of America; 3 Department of Veterinary Pathobiology, College of Veterinary Medicine, University of Missouri, Columbia, Missouri, United States of America; University of Minnesota Hormel Institute, UNITED STATES

## Abstract

Attenuated *Salmonella typhimurium* injected in the circulatory system of mammals selectively targets tumors. Using weekly intraperitoneal injections of attenuated *Salmonella* strain CRC2631, we tested for regression and/or inhibition of tumor development in the TRAMP prostate tumor mouse model, which utilizes SV40 early region expression for autochthonous formation of prostate tumors that progress into metastatic, poorly differentiated prostatic carcinomas in an immunocompetent murine model. Thirteen weekly intraperitoneal administrations of 10^5^–10^7^ CFU CRC2631 into 10 week old mice were well tolerated by the TRAMP model. Sacrifice and histological analysis of TRAMP prostates at 22 weeks indicated that *Salmonella* monotherapy at administrated levels decrease visible tumor size (>29%) but did not significantly inhibit previously described SV40 expression-driven TRAMP tumor progression to undifferentiated carcinomas when histologically examined. In conclusion, this work demonstrates baseline results for CRC2631 *Salmonella* monotherapy using the immunocompetent TRAMP prostate tumor model in preparation for study of combination therapies that resolve autochthonously generated TRAMP prostate tumors, further reduce tumor size, or inhibit prostate tumor progression.

## Introduction

Combatting advanced and metastatic tumors is still one of the most difficult cancer treatment challenges and new therapeutic approaches are necessary to target this heterogeneous disease in which different cancer cells may require different treatments. Targeting the entire cancer cell population within solid tumors is a goal that may be achievable using attenuated bacterial strains, specifically *Salmonella enterica* serovar Typhimurium (*Salmonella*) that preferentially target and infiltrate tumors without affecting non-cancerous cells and tissue [1–3].

Previous research has shown the feasibility of this approach [4–8]. However, high-dosage monotherapy with attenuated *Salmonella* strains proved too toxic and resulted in patients not tolerating the high amounts of attenuated *Salmonella* strains used in clinical studies [9]. These early results indicated that strain modifications as well as determining tolerable doses of *Salmonella* are essential for utilizing this promising therapy to control cancer. Due to direct tumor targeting by *Salmonella* it will also be possible to apply combination therapies by which a drug or cancer-destroying component is directly carried into the cancer cells by *Salmonella* for direct chemotherapeutic administration.

The idea of using bacteriotherapy for treatment of cancer was originally proposed more than a century ago, when heat-killed bacteria and their components were found to have the potential to inhibit cancer growth [10]. In the mid-1900s, it was observed that some bacteria had the ability to survive and replicate in hypoxic tumor tissues. In the last two decades, investigation of bacterial based tumor therapy (bacteriotherapy) has progressed rapidly. Bacterial species including *Salmonella* [11–14], *Listeria* [15, 16], and *Clostridium* [17, 18] have tumor targeting and tumor-destroying phenotypes that are being actively exploited for detection of and chemotherapeutic delivery to tumors [19].

Attenuated *Salmonella* candidates have been extensively studied for targeted treatment of cancer [11]. *Salmonella* are gram-negative facultative anaerobic bacteria that can grow and replicate inside host cells. *Salmonella* strains preferentially infiltrate and colonize solid tumor masses [2, 8] including autochthonous primary or implanted orthologous tumors in the prostate [20], lymph nodes [21], pancreas [22], breast [23], lung [24] and brain tissues [25, 26]. Although the mechanism(s) of *Salmonella* tumor colonization have not been fully elucidated, the *Salmonella* pathogenicity island 2 (SPI-2) is required for rapid amplification of *Salmonella* in tumor host cells [27, 28] that leads to tumor growth suppression [29, 30]. *Salmonella* genetic tools are robust and attenuated strains can be engineered to carry and/or synthesize chemotherapeutic payloads. Finally, *Salmonella* is an adjuvant that can assist in immunogenic recognition [27] and subsequent destruction of tumors [31–33], especially when used in combination with vaccines [34, 35]. The single phase I human clinical trial of a *Salmonella* strain (VNP20009) in human patients had excessive toxicity when used as a cancer monotherapy at high administration levels [9]. Subsequently, research on *Salmonella* as a bacteriotherapeutic has focused on engineering *Salmonella* strains with lowered toxicity [36] while preserving their unique tumor targeting and infiltration phenotypes [3, 37–39]. Delivery of chemotherapeutic payloads is designed to further reduce the *Salmonella* load needed for clinical effect and complete resolution of tumors.

Several *Salmonella* strains are being actively developed as bacteriotherapeutic vectors including VNP20009 [1], A1-R [23], SL7207 [40], LVR01 [41], and CRC2631 [6]. We have developed the tumor-targeting *Salmonella* strain model and candidate therapeutic (CRC2631) that is derived from the *Salmonella typhimurium* LT2 wild type [14]. The parental strain (CRC1674) was stored in agar stabs under nutrient-limiting conditions for more than four decades at room temperature, generating dramatic genetic diversity including deletions, duplications, frameshifts, inversions and transpositions [42, 43]. Genetic investigation indicates CRC1674 contains numerous mutations: originally an LT2 *his*-2550 strain, CRC1674 acquired a *his* suppressor mutation, DIIR49B, an altered *rpoS* start signal (UUG), G to T mutation in position 168 in *rpoS* sequence, and decreased HPI and HPII [14]. CRC1674 was further engineered to disrupt *aroA*, *thyA*, and *rfaH* to generate an LPS-deficient strain auxotrophic for biosynthesis of aromatic amino acids and thymine. The resulting attenuated strain, CRC2631, did not change its tumor targeting and tumor cell destruction phenotype but decreased its toxicity dramatically. Co-incubation of CRC2631 and human prostate cancer cell line PC-3M results in colonization of PC-3M and destruction of their mitochondria within one hour [6]. Up to 1.2 × 10^8^ CFU of CRC2631 can be tolerated in TRAMP mice (an immunocompetent autochthonous prostate cancer model), showing its safety in mammalian hosts. When intraperitoneally injected with 1×10^7^ CFU, the ratio of *Salmonella* counts were up to 100-fold greater in the TRAMP mouse prostate tumor masses versus the usual *Salmonella* reservoirs of the liver and spleen after 72 hours. We have performed morphologic and phenotypic analysis of CRC2631 species recovered from TRAMP mouse prostate tumors to evaluate the selective pressures of cancer targeting and persistence in the novel tumor environment [44].

In order to investigate the ability of CRC2631 to serve as a chemotherapeutic carrying vector, we have explored its effect as a monotherapy in the TRAMP mouse, an autochthonous prostate cancer model triggered by testosterone driven SV40 large and small T-antigen expression [45]. The TRAMP prostate cancer model was chosen due to its autochthonous tumor generation, well-characterized tumor progression stages, and immunocompetency [46]. Male TRAMP mouse prostate tumor progression from 8–24 weeks of age proceeds from spontaneous prostatic intraepithelial neoplasia (PIN), to well-differentiated carcinomas (WDC), Phylloides-like lesions (PHY), and finally poorly differentiated carcinoma (PDC) [47–49]. In addition to testing for increased survival time and tumor size inhibition, measuring inhibition of tumor progression of the TRAMP prostate tumor model is also possible; TRAMP prostate tumors have been partially inhibited from progressing to the late stage WDC and PDC development by up to 81% using plant-derived botanical compounds that have been shown to inhibit the Hedgehog signal pathway [50] as well as limited inhibition of WDC incidence when fed the phytoestrogen genistein [49]. This demonstrates that the TRAMP model is excellent for relatively rapid analysis of primary tumor inhibition at varying mono- or multivalent therapy dosages with simultaneous analysis of adverse immunological effects.

In this paper, we report the effect of weekly intraperitoneal *Salmonella* injections on TRAMP mouse survival, tumor size, and progression. Groups of male TRAMP mice positive for SV40 antigen expression were intraperitoneally injected with 10^5^–10^7^ of *Salmonella* strain CRC2631 or a control buffer injection weekly from 10–22 weeks of age. Survival curve analysis was performed during this injection period. After the 22^nd^ week, surviving TRAMP models were sacrificed and the urogenital tracts extracted to measure visible tumor volumes and perform histological grading of prostate and any visible tumors. Our results show that increasing CRC2631 *Salmonella* monotherapy is well tolerated in the TRAMP model and when given during the 10–22 week tumor development window, decreases the size of visible prostate-associated tumors although under our current experimental conditions it does not prevent tumor progression in the prostate tumor model. This study shows that the TRAMP model is excellent for studying the effect of *Salmonella*-mediated cancer targeted combination therapies including delivery of cancer-inhibiting molecules, generation of anti-cancer peptides or triggering immunostimulatory reactions at the tumor site using *Salmonella*-mediated cancer targeting.

## Materials and Methods

### Bacterial Strain Culturing and Preparation

*Salmonella* strain CRC2631 (derived from nutrient-limited LT2 auxotroph CRC1674 [51]) was used in this study. See Table 1 for complete strain information. All *Salmonella* were grown on nutrient Luria-Bertani (LB) agar plates (25g/L LB powder (Fisher BioReagents), 15 g/L agar (Fisher BioReagents) in deionized water) supplemented with 200 μg/mL thymine (Acros Organics) at 37°C overnight. Strains were cultured in liquid medium by stab inoculating 10mL LB broth (25g/L LB powder in deionized water supplemented with 200 μg/mL thymine in sterile 50mL tubes (Thermo Scientific) with isolated colonies and incubating in a 37°C dry shaker for 16–20 hours.

**Table 1:** Bacterial Strains and Animal Models.

Organism	Genotype	Reference
*Salmonella enterica* serovar Typhimurium		
CRC1674	LT2 *hisD*2550 *rpoS*	Sutton et al (2000)
CRC2631	CRC1674 *aroA*::Tn10TcΔ*rfaH* Δ*thyA*::pKD4	Zhong et al (2008)
*Mus musculus*		
TRAMP	C57BL6/J-PBTag+	Greenberg et al (1995), Sluzarz et al (2010)

Overnight cultures of *Salmonella* CRC2631 grown for injection into mouse models were washed with sterile PBS, normalized to 10^8^ colony forming units (CFU)/mL and diluted appropriately to administer 10^5^, 10^6^, and 10^7^ CFU in 100μl of sterile PBS. Samples were loaded in sterile 25G 1mL TB syringes (BD) and kept at 4°C until injection.

### TRAMP Mouse Studies

All experiments utilized the TRansgenic Adenocarcinoma of Mouse Prostate (TRAMP) C57BL6/J-PBTag+ mouse model (Table 1), originally developed by using the prostate-specific rat probasin promoter (PB) to drive expression of the oncogenic simian virus 40 large tumor antigen-coding region (Tag) [45]. The autochthonous prostate tumor formation and progression in the TRAMP model is well established and considered suitable for use in prostate cancer studies of tumor progression and prevention [46] [49].

Male TRAMP mice were raised on site at the University of Missouri (Columbia, MO) as previously described [50]. University of Missouri institutional guidelines for animal care and use were followed. Mice were housed in pathogen-free microisolator-type cages with wood shaving bedding at 70–75°F, 35–65% humidity with a 12 hour day/night cycle. Mice were free-fed with 5001 Laboratory Rodent diet (LabDiet) and water. Three different concentrations (10^5^,10^6^, and 10^7^ CFU) of viable *Salmonella* (CRC2631) in 100μl PBS were intraperitoneally injected each week for 12 weeks into one of three groups of twenty TRAMP mice from 10–22 weeks of age. A fourth control group was intraperitoneally injected with 100μl sterile PBS as a negative control. Animals were euthanized at the study endpoint (end of 22nd week) following University of Missouri Animal Care and Use Committee standard operating protocols. After euthanasia, the prostate and associated tumor masses were harvested, measured using a caliper, and immediately fixed in Shandon^™^ Formal-Fixx^™^ 10% Neutral Buffered Formalin (Thermo Scientific) overnight at 4°C before harvesting tissues for histological analysis. Two cross section samples (1-2mm) of each fixed prostate sample (or tumor mass if prostate was completely transformed) were paraffin embedded, sectioned (4μm thick sections), mounted on glass slides and stained with hematoxylin and eosin (H&E) for examination and tumor grading by light microscopy.

### Animal Research and Welfare

All animal work was performed at the University of Missouri, Columbia, MO, USA. Experimental protocols and animal husbandry were approved by the University of Missouri Animal Care and Use Committee, Columbia, MO, USA (#7642). On injection days, mice were observed every 2 hours for 8 hours to check for unexpected acute reactions to dosage. On non-injection days, mice were examined daily. During observations a pain/distress evaluation was performed for each of the mice as recommended by the University of Missouri Standard Policy on Painful or Distressful Procedures (University of Missouri IACUC, July 2006) to detect signs of toxicity (e.g. no interest in cage exploration, excess Harderian gland secretions, loss of coordination, over 10% body weight loss, loss of appetite, difficulty breathing). Mice with a score >1.0 on the toxicity and discomfort scale or exhibiting obvious signs of distress were taken and humanely euthanized (S1 File). Animals were euthanized (CO_2_ inhalation for 10 minutes followed by cervical dislocation to ensure euthanasia) at the study endpoint (end of 22nd week) following University of Missouri Animal Care and Use Committee standard operating protocols. During the study, there was one unexpected combat death between mice. The remaining deaths in the study were due to TRAMP prostate tumor development (natural morbidity of the mouse line). On-site veterinarian staff administered analgesics as needed.

### Histology

Each prostate was sampled twice. One tissue section per slide was viewed and graded. The veterinary pathologist was unaware of the duplicate slides or treatment groups until after grading. Dorsal prostate tubules were graded individually and placed into one of six categories: 1) normal tissue, 2) hyperplasia (HYP), 3) prostatic intraepithelial neoplasia (PIN), 4) well differentiated carcinoma (WDC), 5) phylloides-like (PHY) and 6) neuro-endocrine-like / poorly differentiated carcinoma (PDC) phenotype as previously described [49]. Identification of neuroendocrine-like poorly differentiated carcinoma (PDC) lesions caused a stage of PDC to be assigned to the animal regardless of the status of the dorsal prostate. Anterior prostate tubules were also viewed and recorded as displaying hyperplasia or well-differentiated adenocarcinoma but not specifically counted or graded. When appropriate, tumors were recorded as localized to a specific section of tissue (i.e. periurethral region) or as affecting the entire tissue.

### Statistical Analysis

GraphPad Prism 6 was used to perform analyses (GraphPad, La Jolla, CA). We used the LIFETEST procedure in SAS 9.4 to compute nonparametric estimates of the survival functions and to compare the survival curves. This procedure was used since we had a high presence of right-censored data from terminating the experiment before many mice died in order to collect prostate tissue for histology. To test whether Salmonella injections significantly inhibited tumor progression, we used Fisher’s exact test.

## Results and Discussion

### Weekly intraperitoneal *Salmonella* injections are well tolerated in the TRAMP mouse model

**Fig 1:**
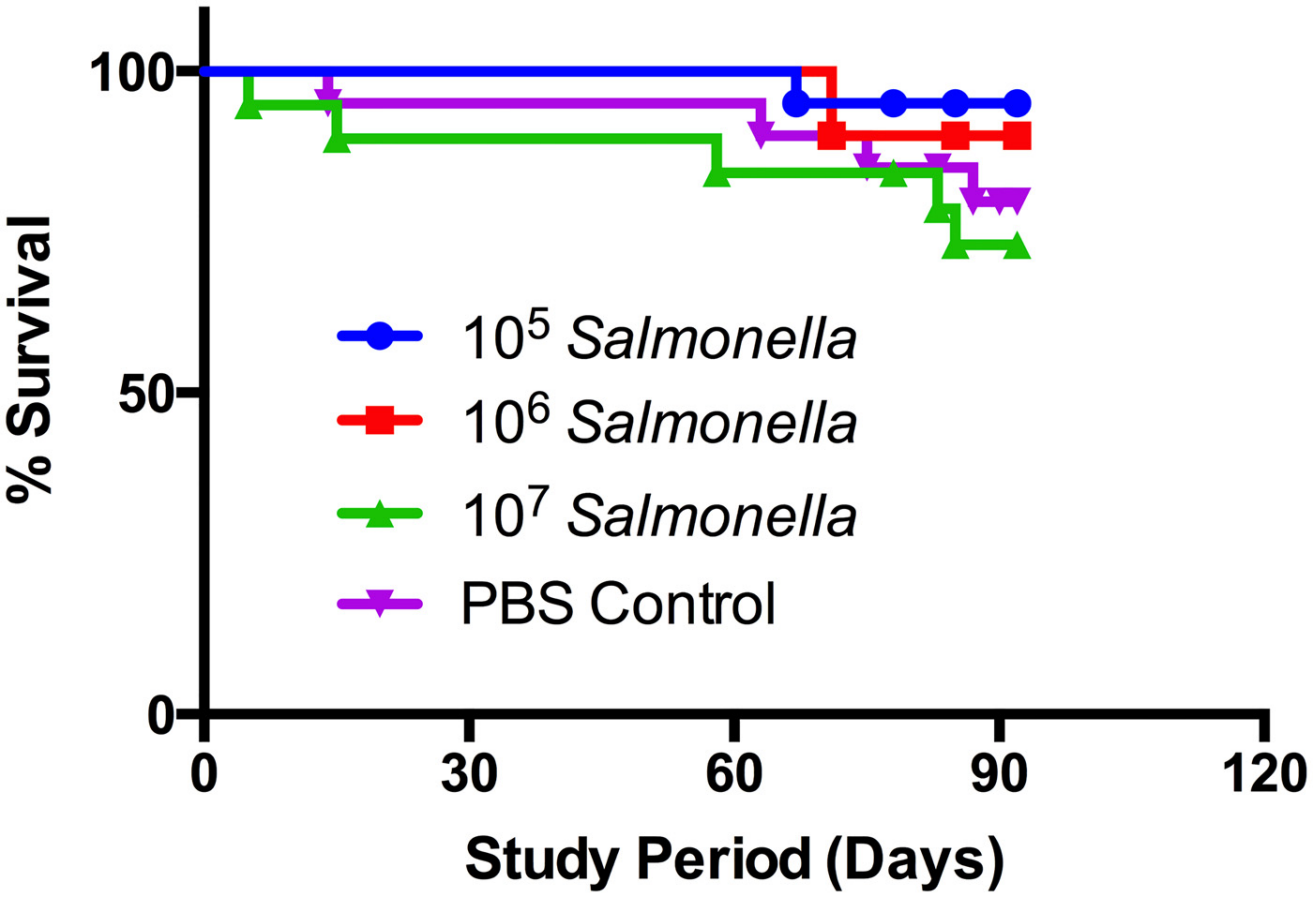
Weekly *Salmonella* injections tolerated by TRAMP mouse model. Survival curve of TRAMP mouse groups (n = 20) aged 10–22 weeks (98 days) during study period with weekly IP injection of 100μl PBS (control) or 100μl PBS with *Salmonella* at indicated concentrations. Surviving mice were sacrificed at the end of week 22.

TRAMP mouse models of autochthonous prostate cancer groups (n = 20) were injected intraperitoneally with 10^5^, 10^6^, and 10^7^ CFU of *Salmonella* strain CRC2631 in 100μl PBS. Intraperitoneal (IP) injections of 100μl PBS were performed in one group as a negative control. Survival curves were plotted during the study period (Fig 1). Survival curves indicate no significant change in survival for the TRAMP model over the study period at any injection level (S1 Fig, S1 Table). One mouse in the 10^7^ group died from combat-associated injuries with cage mates; this death was excluded in the survival curve analysis because we cannot tell if the death was due to combat injuries or tumor burden.

### Average size of tumor volumes in TRAMP model decrease with increased *Salmonella* injection levels

**Fig 2:**
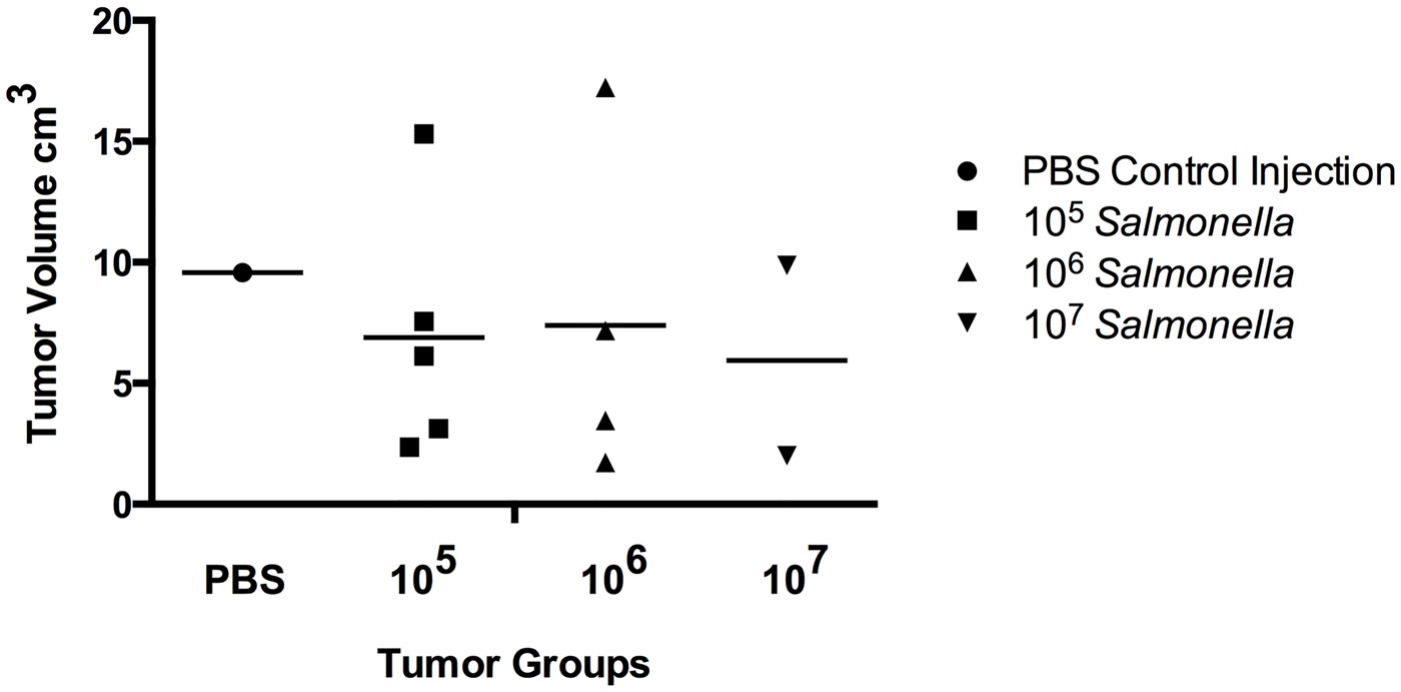
Tumor volume decreases with increasing *Salmonella* dosage. Caliper measurement of prostate-associated tumor volumes extracted from TRAMP mouse groups at end of study with visible excess tumor growth. Mean tumor volumes: PBS control (n = 1/16 with visible excess growth) 9.57 cm^3^, 10^5^ injection group (n = 5/17 with visible excess growth) 7.45 cm^3^, 10^6^ injection group (n = 5/17 with visible excess growth) 6.43 cm^3^, 10^7^ injection group (n = 2/13 with visible excess growth) 5.94 cm^3^.

Prostate and prostate-associated tumors were extracted from surviving TRAMP mice at study endpoint. Volumes of prostate-associated tumor masses were measured using calipers (Fig 2). Mice with no visible tumor masses were not measured. Visible tumor mass mice dosed with *Salmonella* had 29.2% smaller average tumor burdens. Average volume of recovered tumors decreased with increase in *Salmonella* dosage. Due to the single data point in the control group, this data is qualitative.

### Histological grading of prostate tumors in the TRAMP mouse model

**Fig 3:**
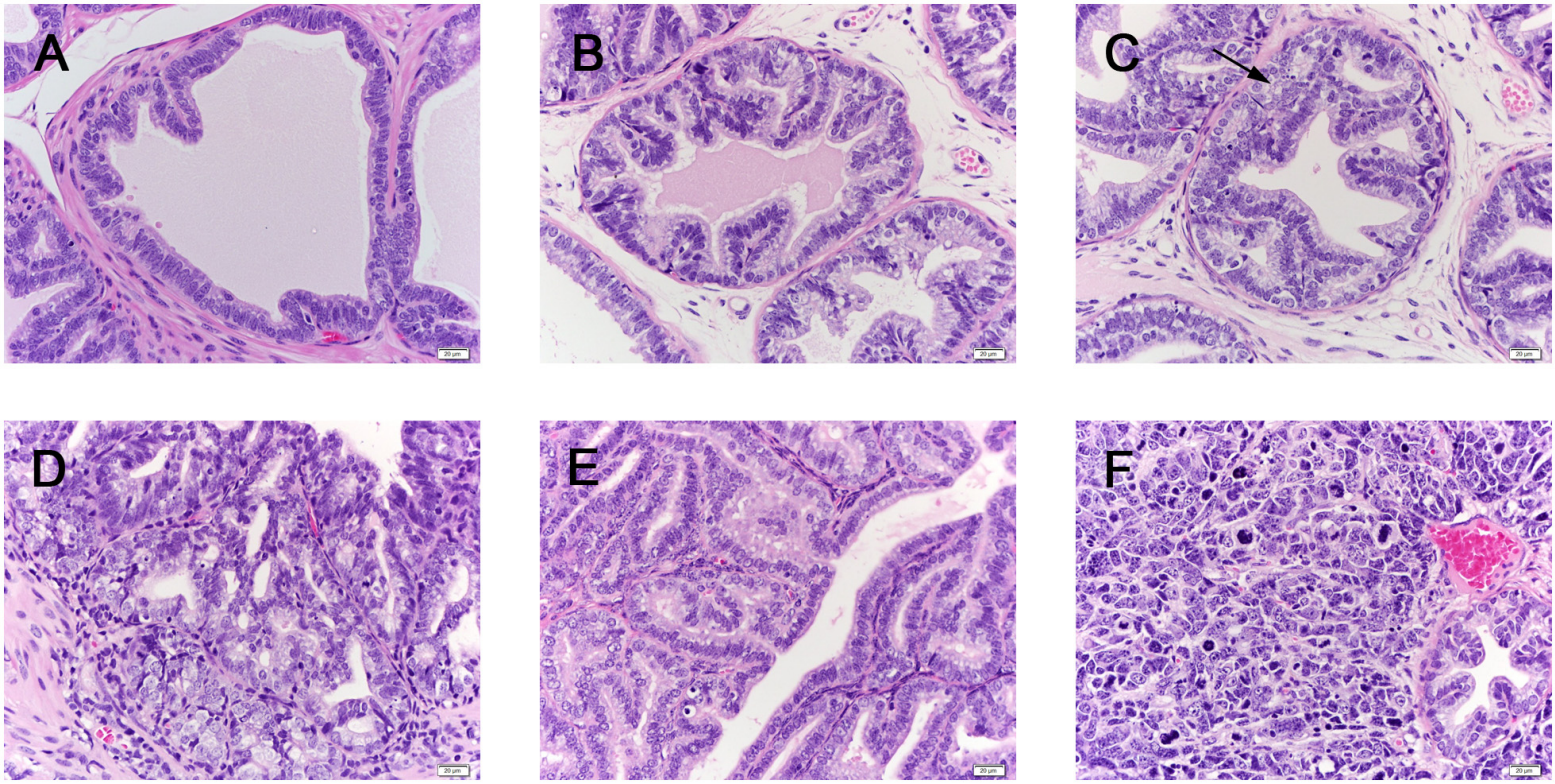
Histology of prostate tumor development in TRAMP mice. Histologic sections of the dorsal lobes of the prostate from transgenic mice stained with hematoxylin and eosin at 40X magnification. **Pathologic grades**: PIN, prostatic epithelial neoplasia; WD, well-differentiated adenocarcinoma; PHY, phylloides-like; PDC, poorly differentiated neuroendocrine-type carcinoma. **Slides**: (A) Normal tissue, (B) Hyperplastic tissue, (C) PIN, (D) WD, (E) PHY and (F) PDC (neuroendocrine-type). **Observations**: (C) Note tufting of epithelial cells, increased mitoses, hyperchromatic nuclei, stratification of nuclei and cribiform structures (arrow). (D) Note neoplastic cells with round nuclei; tumor type is characterized by increased numbers of small glands and thickening of the stroma. (E) Note staghorn luminal patterns of neoplastic cells. (F) Note the high nuclear:cytoplasmic ratio of neoplastic cells, loss of glandular differentiation and marked cell pleomorphism.

**Fig 4:**
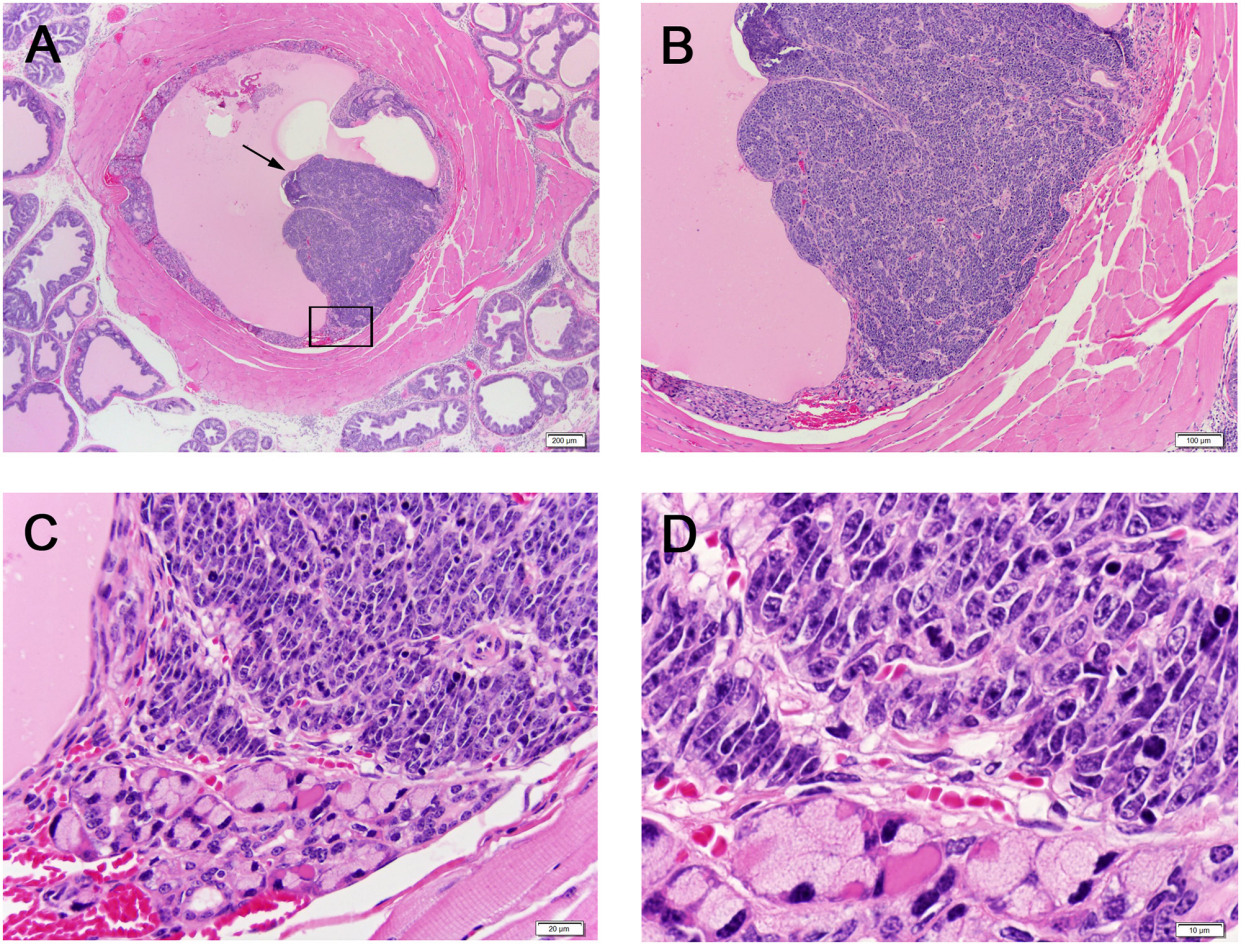
Neuroendocrine-type carcinoma in the periurethral region of a TRAMP mouse. Histologic sections of the periurethral region from a transgenic mouse stained with hematoxylin and eosin (H&E) at 4X (A), 10X (B), 40X (C) and 100X (D). (A): Note discrete tumor (arrow) within the epithelium of the periurethral region. The outline in (A) is the magnified region shown in (C) and (D).

Prostates along with any associated tumors were extracted from the surviving TRAMP mouse models and fixed overnight in 10% buffered formalin at 4°C. Two cross sections of the dorsal prostate and any associated tumors were sampled by sectioning, hematoxylin and eosin staining, and grading using previously established criteria [49]. Histological grading of *Salmonella*-treated prostates ranged from normal tissue to poorly differentiated carcinomas (Fig 3). Comparing tumor stages individually, using Chi-square test for association for PDC and Fisher’s exact test for PHY and WD (because some cell sizes are smaller than 5), The p-values were all non-significant: PDC p-value = 0.1725, PHY p-value = 0.3967, WD p-value = 0.4458. Overall association between the injection groups and tumor progression is not statistically significant (P-value = 0.3314) using Fisher’s exact test. Therefore, *Salmonella* injections did not significantly inhibit tumor progression in the TRAMP prostate cancer model (Table 2). Histological observation indicated the presence of neuroendocrine-type tumors at the periurethral region in twelve TRAMP prostate samples (example, Fig 4). While neuroendocrine tumors in the TRAMP model have been previously reported to invade the periurethral region, this has previously been reported as always associated with a morphologically identical large tumor arising in the prostate [47]; we only observed large neuroendocrine tumors associated with two of the twelve samples.

**Table 2:** Effect of weekly Salmonella dosages on prostate tumor development in the 5 month TRAMP prostate.

		Farthest tumor progression in prostate		
*Salmonella* Dosage	n	WD	PHY	PDC
PBS Control	16	4	5	7
10^5^ *Salmonella*	17	2	3	12
10^6^ *Salmonella*	17	6	5	6
10^7^ *Salmonella*	13	4	1	8

Numbers indicate the farthest stage of progression for each prostate sample. n = sample size. WD, well-differentiated adenocarcinoma; PHY, phylloides-like carcinoma; PDC, poorly differentiated neuroendocrine-type carcinoma.

Prostate cancer is still the second-leading cause of cancer-related deaths in men [52]. Advanced cancer treatment still represents medical challenges, as effective cures are still not yet available. While androgen deprivation therapy (ADT) is effective in treating early stages of advanced prostate cancer most patients respond to this treatment initially but their cancers become androgen-independent and most patients become ADT resistant [53, 54].

Furthermore, a surprising emergence of neuroendocrine prostate cancer cells (NEPC) has resulted from androgen deprivation therapy (ADT), which presents new challenges for treatment. While the AR-negative neuroendocrine prostate cancers (NEPC) are rare at the time of initial diagnosis, they can account for 5–30% of advanced prostate cancers promoted by ADT [55].

Effective therapy for advanced stages of androgen-independent prostate cancer is still not yet available. Although progress has been made toward identifying the problems associated with disease progression, it has become clear that there is a need to eradicate the various subpopulations of this heterogeneous disease including the hard-to-treat and oftentimes radiation-resistant cancer stem cell (CSC) population. New therapies are critically needed to target these subpopulations that may require different and combination treatment strategies.

While rigorous and targeted therapies for the various subpopulations of prostate cancer might be developed in the distant future new treatment options aimed at targeting the entire cancer tissue with all subpopulations are currently in development and actively pursued in various laboratories using different approaches. A number of laboratories, including ours, are utilizing attenuated bacteria for targeting and chemotherapeutic activation/delivery (“bacteriotherapy”) of cancers. These approaches are expected to have advantages over surgery and radiotherapy and will also eliminate newly observed cancer stem cell populations that have presented new challenges for treatment of cancer, as the inability to provide new treatment options is still associated with poor prognosis. The cancer stem cell subpopulation is responsible for prostate tumor initiation, recurrence, drug-resistance and metastatic progression. *Salmonella* targeting significantly reduces the weight of tumors initiated by cancer stem-like cells in several studies [56, 57].

*Salmonella* have been shown to effectively target and colonize any tumors that can be accessed by the host circulatory system, whether the *Salmonella* is introduced by intravenous, intraperitoneal, or oral delivery [11, 12]. *Salmonella* adapted to target tumors for detection have been predicted to colonize and detect tumor masses more than 2000-fold smaller than current tumor detection methods utilizing tomography [19].

Evolved to survive in mammalian hosts, *Salmonella* has the ability to adapt host membrane vesicles for its own use and can manipulate the placement of the membrane vesicle for replication and infiltration (invasion) of adjacent host cells [27]. As a facultative anaerobe, *Salmonella* can colonize both the oxygen-rich tumor periphery and anoxic tumor mass [8, 38, 58]. Once the entire tumor is colonized, *Salmonella* can deliver attached chemotherapies or synthesize molecules including cancer-killing chemotherapeutics, enzymes for activating drugs (prodrugs) at the cancer site [59, 60], transfer and/or express genes to inhibit cancer oncogene expression, or produce immune signaling molecules for cancer immunotherapy [35]. The high infiltration rate of *Salmonella* makes it superior to current nanoparticle technology that is limited in how far it can penetrate tumor tissue due to the high interstitial pressure characteristic of tumor masses [61]. Novel combination bacteriotherapies including targeted delivery of anti-cancer molecules (carried or synthesized), immunostimulatory peptides or vaccines, enzymes designed to activate prodrugs at tumors, and radiation [62] combination therapies, all concentrated at the tumor site using *Salmonella*, have been and continue to be actively researched by our laboratory and other laboratories in the field of cancer-targeting bacteriotherapy with increasing levels of success.

The single limitation to *Salmonella* bacteriotherapy is concern about potential toxicity seen in cancer patients using the VNP20009 *Salmonella* strain during phase I clinical testing in 2002 [9]. Since that study, efforts have been made by multiple laboratories to reduce toxicity in *Salmonella* without disrupting its cancer targeting, invasion, and tumor infiltration phenotypes. We engineered a novel, attenuated *Salmonella* bacteriotherapeutic strain (CRC2631) that is non-toxic and exhibits cancer targeting, invasion, and cancer cell destruction phenotypes [6]. Additionally, we have developed tools to facilitate *Salmonella* vector delivery of combination chemotherapies in order to increase bacteriotherapeutic effectiveness and reduce the dose of *Salmonella* needed for clinical effect [63].

The effect of *Salmonella* monotherapy on prostate tumor progression in an immunocompetent model has not been characterized. In the present study, we examined the effect of weekly administration of our bacteriotherapeutic *Salmonella* strain (CRC2631) on prostate tumor progression. We used the TRAMP mouse model of prostate cancer that utilizes testosterone-driven expression of the SV40 large and small T-antigen [45] to generate autochthonous primary prostate tumors that eventually develop into poorly differentiated carcinomas with neuroendocrine carcinomas. Tumor progression in the TRAMP model is well documented [49, 50, 64] and provides an excellent model to test prostate tumor progression inhibition of *Salmonella* monotherapy and combination chemotherapies in an immunocompetent mammalian model that translates well to study prostate cancer progression in human patients.

We have shown that *Salmonella* CRC2631 injections in the immunocompetent TRAMP model are well tolerated; survival curves of TRAMP mice during the 10–22 week injection period show no significant decrease in survival in TRAMP mice during weekly IP injections of 10^5^−10^7^ CFUs of *Salmonella* versus control injections of sterile PBS. Secondly, the mean size of visible prostate-associated tumors observed in TRAMP mice decreased when *Salmonella* was administered; as more *Salmonella* was administered, the average size of prostate-associated tumors also decreased. Due to the single data point in the PBS control group (Fig 2) we cannot state with confidence that CRC2631 caused significant reduction of tumor size; however this qualitative data in combination with the reduction of TRAMP mice at the final PDC stage of SV40 expression-induced prostate cancer in the 10^6^ group (Table 2) suggests that CRC2631 Salmonella monotherapy is reducing their tumor burden in the models with excess tumor growth and increasing their quality of life. These subtle but promising results with CRC2631 Salmonella monotherapy make the immunocompetent, autochthonous TRAMP prostate cancer progression model an excellent candidate for evaluating combination therapies, including but not limited to inhibitors of the Hedgehog signaling pathway which has previously shown partial inhibition of TRAMP tumor progression [50]. The conclusion that Salmonella bacteriotherapy requires additional carried and/or expressed anti-cancer molecules delivered by tumor-infiltrated *Salmonella* (combination therapy) is a commonly held opinion by prominent researchers in the field of bacteriotherapy [12, 13, 36, 65].

In humans and mice the normal prostate is composed of stromal and epithelial compartments. The epithelial compartment contains luminal epithelial cells, basal cells and a few scattered neuroendocrine (NE) cells. NE cells have epithelial, neural and endocrine features. They are not evenly distributed in the prostate and are most often found in the periurethral region and verumontanum (colliculus seminalis) in humans [66]. NE cells can also be found in prostate cancer, with increased numbers of these cells in tumors associated with poor prognosis [66].

In humans, the term neuroendocrine differentiation (NED) in prostate cancer (PC) refers to the presence of singly scattered NE cells or cells in small nests in typical prostatic adenocarcinomas [66]. Focal neuroendocrine differentiation is common in human prostatic adenocarcinoma [67]. NED is seen in >30% of prostate cancer and is associated with poor prognosis (high grade and high stage tumors) and androgen independence [68]. About 5–10% of prostatic adenocarcinomas contain large numbers of NE tumor cells, however, pure NE tumors in humans are rare as primary cancers [66].

As in human neuroendocrine PC, neuroendocrine carcinomas in TRAMP mice are associated with rapid growth and metastases and are highly lethal. However, while only a small percentage of human prostate tumors are primary NE cancers, TRAMP mice have a high incidence of neuroendocrine tumors arising in the prostate, which often metastasize to the lymph nodes, lung and liver [67]. In mice, neuroendocrine carcinomas are similar to human neuroendocrine carcinomas in appearance and are characterized by cells with high nuclear:cytoplasmic ratio of neoplastic cells, granular cytoplasm, loss of glandular differentiation and marked cell pleomorphism. They are the most widely metastatic and aggressive mouse prostate cancer [67]. The TRAMP model is therefore also suitable to study NED.

Based on histology analysis and grading, we did not find a significant reduction in tumor progression in the TRAMP model using *Salmonella* monotherapy. However, we had an unexpected and novel finding. Histological observation indicated the presence of neuroendocrine-type tumors at the periurethral region in twelve TRAMP prostate thick sections. While neuroendocrine tumors in the TRAMP model have been previously reported to invade the periurethral region, this has previously been reported as always associated with a morphologically identical large tumor arising in the prostate [47]; we only observed large neuroendocrine tumors associated with two of the twelve samples. We are quite confident that there are no larger tumors in the rest of the ten prostates with neuroendocrine-type tumors at the periurethral region, which is a novel observation in the TRAMP model. However, we cannot eliminate the possibility that there are small neuroendocrine-type tumors in the prostate because we did not perform serial sections of the ten prostates that did not have visible large tumors. Invasion of neuroendocrine type tumor cells at the periurethral region in the TRAMP model is important, as it demonstrates the utility of this model to study neuroendocrine type tumor cells that have become an important aspect for the treatment of aggressive prostate tumors. As indicated above, in human prostate cancer androgen deprivation therapy (ADT) is commonly used for treatment of prostate cancer, which is associated with promoting the progression of androgen receptor (AR)-positive adenocarcinoma cells (AdPC) to AR negative neuroendocrine prostate cancer (NEPC) through neuroendocrine differentiation (NED). However, treating NEPC is difficult, as no potent drugs are available for this type of cancer progression. We plan to follow up and investigate the effect of *Salmonella* bacteriotherapy on neuroendocrine type tumor cells.

## Conclusions

In summary, in this study we showed that weekly injections of 10^5^−10^7^ of bacteriotherapeutic *Salmonella* strain CRC2631 in the TRAMP mouse prostate tumor progression model during tumor development over weeks 10–22 are well tolerated and increased administration of *Salmonella* results in smaller prostate-associated tumors in the mouse groups that reached study endpoint; however, TRAMP tumor progression was unaffected by administration of CRC2631 *Salmonella* monotherapy. Additionally, we observed multiple instances of neuroendocrine-type tumor tissue at the periurethral region in TRAMP prostate cancer mice, associating the TRAMP prostate cancer model with more aggressive androgen receptor negative neuroendocrine human prostate cancers seen after androgen deprivation therapy in human patients.

Using this baseline data, investigators can now proceed to studies employing *Salmonella*-delivered combination chemotherapies in the immunocompetent, autochthonous, neuroendocrine TRAMP prostate tumor model and look for improved tumor resolutions with different types and concentrations of *Salmonella*-delivered combination chemotherapies.

## Supporting Information

S1 Fig Statistical survival curve analysis. The top graph shows the survival curves for all four groups with 95% confidence intervals. The bottom table is the product-limit survival estimates for the four groups without 95% confidence intervals.
(PDF)

S1 File Pain/distress evaluation for rodents. TRAMP mice that scored 1.0 or greater on the pain/distress evaluation were euthanized after consultation with the attending veterinarian.
(PDF)

S1 Table Statistical analysis of survival data between *Salmonella* injection groups. The Type 3 Tests for differences between the four groups gives a Wald Chi-Square of 2.6188 with 3 degrees of freedom (DF) and corresponds with a p-value of 0.4542. This tells us that there is not sufficient evidence to conclude that there are any differences in survival among the four groups. Further, we can look at the Analysis of Maximum Likelihood Estimates to see the Hazard Ratio (compared to the control group) and the p-values for comparison to the control group. All p-values are greater than 0.05 so we conclude that there is not sufficient evidence to conclude that any of the groups (10^5, 10^6, 10^7) have significantly different survival than the control group.
(PDF)

## References

[1] Pawelek JM, Low KB, Bermudes D. Tumor-targeted Salmonella as a novel anticancer vector. Cancer Res. 1997;57(20):4537–44. Epub 1997/10/23. . 9377566

[2] Forbes NS, Munn LL, Fukumura D, Jain RK. Sparse initial entrapment of systemically injected Salmonella typhimurium leads to heterogeneous accumulation within tumors. Cancer Res. 2003;63(17):5188–93. . 14500342

[3] Toley BJ, Forbes NS. Motility is critical for effective distribution and accumulation of bacteria in tumor tissue. Integrative biology: quantitative biosciences from nano to macro. 2012;4(2):165–76. Epub 2011/12/24. 10.1039/c2ib00091a . 22193245 PMC4956405

[4] Pawelek JM, Low KB, Bermudes D. Bacteria as tumour-targeting vectors. Lancet Oncol. 2003;4(9):548–56. . 12965276 10.1016/s1470-2045(03)01194-x

[5] Zhao M, Geller J, Ma H, Yang M, Penman S, Hoffman RM. Monotherapy with a tumor-targeting mutant of Salmonella typhimurium cures orthotopic metastatic mouse models of human prostate cancer. Proc Natl Acad Sci U S A. 2007;104(24):10170–4. Epub 2007/06/06. 10.1073/pnas.0703867104 17548809 PMC1891231

[6] Zhong Z, Kazmierczak RA, Dino A, Khreis R, Eisenstark A, Schatten H. Salmonella-host cell interactions, changes in host cell architecture, and destruction of prostate tumor cells with genetically altered Salmonella. Microsc Microanal. 2007;13(5):372–83. Epub 2007/09/29. 10.1017/S1431927607070833 . 17900389

[7] Ganai S, Arenas RB, Forbes NS. Tumour-targeted delivery of TRAIL using Salmonella typhimurium enhances breast cancer survival in mice. British journal of cancer. 2009;101(10):1683–91. Epub 2009/10/29. 10.1038/sj.bjc.6605403 19861961 PMC2778534

[8] Leschner S, Westphal K, Dietrich N, Viegas N, Jablonska J, Lyszkiewicz M, et al Tumor invasion of Salmonella enterica serovar Typhimurium is accompanied by strong hemorrhage promoted by TNF-alpha. PloS one. 2009;4(8):e6692 Epub 2009/08/21. 10.1371/journal.pone.0006692 19693266 PMC2724709

[9] Toso JF, Gill VJ, Hwu P, Marincola FM, Restifo NP, Schwartzentruber DJ, et al Phase I study of the intravenous administration of attenuated Salmonella typhimurium to patients with metastatic melanoma. J Clin Oncol. 2002;20(1):142–52. Epub 2002/01/05. 11773163 10.1200/JCO.2002.20.1.142 PMC2064865

[10] Coley W. The cancer symposium at Lake Mohonk. Am J Surg. 1929;1:22–3.

[11] Leschner S, Weiss S. Salmonella-allies in the fight against cancer. J Mol Med (Berl). 2010;88(8):763–73. Epub 2010/06/08. 10.1007/s00109-010-0636-z . 20526574

[12] Forbes NS. Engineering the perfect (bacterial) cancer therapy. Nat Rev Cancer. 2010;10(11):785–94. Epub 2010/10/15. 10.1038/nrc2934 . 20944664 PMC3756932

[13] Hoffman RM. Back to the Future: Are Tumor-Targeting Bacteria the Next-Generation Cancer Therapy? Methods in molecular biology (Clifton, NJ. 2015;1317:239–60. 10.1007/978-1-4939-2727-2_14 . 26072411

[14] Eisenstark A, Kazmierczak RA, Dino A, Khreis R, Newman D, Schatten H. Development of Salmonella strains as cancer therapy agents and testing in tumor cell lines. Methods in molecular biology (Clifton, NJ. 2007;394:323–54. Epub 2008/03/28. 10.1007/978-1-59745-512-1_16 . 18363243

[15] Paterson Y, Guirnalda PD, Wood LM. Listeria and Salmonella bacterial vectors of tumor-associated antigens for cancer immunotherapy. Seminars in immunology. 2010;22(3):183–9. Epub 2010/03/20. 10.1016/j.smim.2010.02.002 . 20299242 PMC4411241

[16] Quispe-Tintaya W, Chandra D, Jahangir A, Harris M, Casadevall A, Dadachova E, et al Nontoxic radioactive Listeriaat is a highly effective therapy against metastatic pancreatic cancer. Proc Natl Acad Sci U S A. 2013 Epub 2013/04/24. 10.1073/pnas.1211287110 . 23610422 PMC3666740

[17] Theys J, Pennington O, Dubois L, Anlezark G, Vaughan T, Mengesha A, et al Repeated cycles of Clostridium-directed enzyme prodrug therapy result in sustained antitumour effects in vivo. British journal of cancer. 2006;95(9):1212–9. Epub 2006/10/07. 10.1038/sj.bjc.6603367 17024128 PMC2360559

[18] Roberts NJ, Zhang L, Janku F, Collins A, Bai RY, Staedtke V, et al Intratumoral injection of Clostridium novyi-NT spores induces antitumor responses. Science translational medicine. 2014;6(249):249ra111 10.1126/scitranslmed.3008982 25122639 PMC4399712

[19] Panteli JT, Forkus BA, Van Dessel N, Forbes NS. Genetically modified bacteria as a tool to detect microscopic solid tumor masses with triggered release of a recombinant biomarker. Integrative biology: quantitative biosciences from nano to macro. 2015;7(4):423–34. 10.1039/c5ib00047e 25737274 PMC4390529

[20] Fensterle J, Bergmann B, Yone CL, Hotz C, Meyer SR, Spreng S, et al Cancer immunotherapy based on recombinant Salmonella enterica serovar Typhimurium aroA strains secreting prostate-specific antigen and cholera toxin subunit B. Cancer gene therapy. 2008;15(2):85–93. Epub 2007/12/18. 10.1038/sj.cgt.7701109 . 18084243

[21] Meng JZ, Dong YJ, Huang H, Li S, Zhong Y, Liu SL, et al Oral vaccination with attenuated Salmonella enterica strains encoding T-cell epitopes from tumor antigen NY-ESO-1 induces specific cytotoxic T-lymphocyte responses. Clin Vaccine Immunol. 2010;17(6):889–94. Epub 2010/04/09. 10.1128/CVI.00044-10 20375244 PMC2884430

[22] Nagakura C, Hayashi K, Zhao M, Yamauchi K, Yamamoto N, Tsuchiya H, et al Efficacy of a genetically-modified Salmonella typhimurium in an orthotopic human pancreatic cancer in nude mice. Anticancer research. 2009;29(6):1873–8. . 19528442

[23] Zhao M, Yang M, Ma H, Li X, Tan X, Li S, et al Targeted therapy with a Salmonella typhimurium leucine-arginine auxotroph cures orthotopic human breast tumors in nude mice. Cancer Res. 2006;66(15):7647–52. Epub 2006/08/04. 10.1158/0008-5472.CAN-06-0716 . 16885365

[24] Shao C, Zhao L, Wang K, Xu W, Zhang J, Yang B. The tumor suppressor gene RBM5 inhibits lung adenocarcinoma cell growth and induces apoptosis. World journal of surgical oncology. 2012;10:160 Epub 2012/08/08. 10.1186/1477-7819-10-160 22866867 PMC3502321

[25] Momiyama M, Zhao M, Kimura H, Tran B, Chishima T, Bouvet M, et al Inhibition and eradication of human glioma with tumor-targeting Salmonella typhimurium in an orthotopic nude-mouse model. Cell cycle (Georgetown, Tex. 2012;11(3):628–32. Epub 2012/01/26. 10.4161/cc.11.3.19116 22274398 PMC3315098

[26] Hoffman RM. Tumor-targeting amino acid auxotrophic Salmonella typhimurium. Amino acids. 2009;37(3):509–21. 10.1007/s00726-009-0261-8 19291366

[27] LaRock DL, Chaudhary A, Miller SI. Salmonellae interactions with host processes. Nature reviews. 2015;13(4):191–205. 10.1038/nrmicro3420 . 25749450 PMC5074537

[28] Chakraborty S, Mizusaki H, Kenney LJ. A FRET-based DNA biosensor tracks OmpR-dependent acidification of Salmonella during macrophage infection. PLoS Biol. 2015;13(4):e1002116 10.1371/journal.pbio.1002116 25875623 PMC4397060

[29] Pawelek JM, Sodi S, Chakraborty AK, Platt JT, Miller S, Holden DW, et al Salmonella pathogenicity island-2 and anticancer activity in mice. Cancer gene therapy. 2002;9(10):813–8. . 12224021 10.1038/sj.cgt.7700501

[30] Szeto J, Namolovan A, Osborne SE, Coombes BK, Brumell JH. Salmonella-containing vacuoles display centrifugal movement associated with cell-to-cell transfer in epithelial cells. Infect Immun. 2009;77(3):996–1007. 10.1128/IAI.01275-08 19103768 PMC2643626

[31] Zhu X, Zhou P, Cai J, Yang G, Liang S, Ren D. Tumor antigen delivered by Salmonella III secretion protein fused with heat shock protein 70 induces protection and eradication against murine melanoma. Cancer science. 2010;101(12):2621–8. Epub 2010/10/01. 10.1111/j.1349-7006.2010.01722.x . 20880334 PMC11159612

[32] Lee CH, Hsieh JL, Wu CL, Hsu PY, Shiau AL. T cell augments the antitumor activity of tumor-targeting Salmonella. Applied microbiology and biotechnology. 2011;90(4):1381–8. Epub 2011/03/02. 10.1007/s00253-011-3180-z . 21360146

[33] Lee CH, Hsieh JL, Wu CL, Hsu HC, Shiau AL. B cells are required for tumor-targeting Salmonella in host. Applied microbiology and biotechnology. 2011;92(6):1251–60. Epub 2011/06/15. 10.1007/s00253-011-3386-0 . 21667275

[34] Hong EH, Chang SY, Lee BR, Pyun AR, Kim JW, Kweon MN, et al Intratumoral injection of attenuated Salmonella vaccine can induce tumor microenvironmental shift from immune suppressive to immunogenic. Vaccine. 2013;31(10):1377–84. 10.1016/j.vaccine.2013.01.006 . 23318147

[35] Chang SY, Kim YJ, Ko HJ. Potential therapeutic anti-tumor effect of a Salmonella-based vaccine. Hum Vaccin Immunother. 2013;9(8):1654–60. 10.4161/hv.24917 23733040 PMC3906262

[36] Frahm M, Felgner S, Kocijancic D, Rohde M, Hensel M, Curtiss R 3rd, et al Efficiency of conditionally attenuated Salmonella enterica serovar Typhimurium in bacterium-mediated tumor therapy. MBio. 2015;6(2). 10.1128/mBio.00254-15 25873375 PMC4453544

[37] Arrach N, Cheng P, Zhao M, Santiviago CA, Hoffman RM, McClelland M. High-throughput screening for salmonella avirulent mutants that retain targeting of solid tumors. Cancer Res. 2010;70(6):2165–70. Epub 2010/03/17. 10.1158/0008-5472.CAN-09-4005 . 20231149 PMC4103738

[38] Ganai S, Arenas RB, Sauer JP, Bentley B, Forbes NS. In tumors Salmonella migrate away from vasculature toward the transition zone and induce apoptosis. Cancer gene therapy. 2011;18(7):457–66. Epub 2011/03/26. 10.1038/cgt.2011.10 21436868 PMC3117926

[39] Thornlow DN, Brackett EL, Gigas JM, Van Dessel N, Forbes NS. Persistent enhancement of bacterial motility increases tumor penetration. Biotechnol Bioeng. 2015 10.1002/bit.25645 . 25976712 PMC4586311

[40] Berger E, Soldati R, Huebener N, Hohn O, Stermann A, Durmus T, et al Salmonella SL7207 application is the most effective DNA vaccine delivery method for successful tumor eradication in a murine model for neuroblastoma. Cancer letters. 2013;331(2):167–73. 10.1016/j.canlet.2012.12.026 . 23337288

[41] Grille S, Moreno M, Bascuas T, Marques JM, Munoz N, Lens D, et al Salmonella enterica serovar Typhimurium immunotherapy for B-cell lymphoma induces broad anti-tumour immunity with therapeutic effect. Immunology. 2014;143(3):428–37. 10.1111/imm.12320 24834964 PMC4212956

[42] Porwollik S, Wong RM, Helm RA, Edwards KK, Calcutt M, Eisenstark A, et al DNA amplification and rearrangements in archival Salmonella enterica serovar Typhimurium LT2 cultures. J Bacteriol. 2004;186(6):1678–82. Epub 2004/03/05. 14996798 10.1128/JB.186.6.1678-1682.2004 PMC355959

[43] Eisenstark A. Genetic diversity among offspring from archived Salmonella enterica ssp. enterica serovar typhimurium (Demerec Collection): in search of survival strategies. Annu Rev Microbiol. 2010;64:277–92. Epub 2010/09/10. 10.1146/annurev.micro.091208.073614 . 20825350

[44] Choe E, Kazmierczak RA, Eisenstark A. Phenotypic evolution of therapeutic Salmonella enterica serovar Typhimurium after invasion of TRAMP mouse prostate tumor. MBio. 2014;5(4):e01182–14. 10.1128/mBio.01182-14 24987088 PMC4161240

[45] Greenberg NM, DeMayo F, Finegold MJ, Medina D, Tilley WD, Aspinall JO, et al Prostate cancer in a transgenic mouse. Proc Natl Acad Sci U S A. 1995;92(8):3439–43. . 7724580 10.1073/pnas.92.8.3439 PMC42182

[46] Grabowska MM, DeGraff DJ, Yu X, Jin RJ, Chen Z, Borowsky AD, et al Mouse models of prostate cancer: picking the best model for the question. Cancer metastasis reviews. 2014;33(2–3):377–97. 10.1007/s10555-013-9487-8 24452759 PMC4108581

[47] Shappell SB, Thomas GV, Roberts RL, Herbert R, Ittmann MM, Rubin MA, et al Prostate pathology of genetically engineered mice: definitions and classification. The consensus report from the Bar Harbor meeting of the Mouse Models of Human Cancer Consortium Prostate Pathology Committee. Cancer Res. 2004;64(6):2270–305. Epub 2004/03/18. . 15026373 10.1158/0008-5472.can-03-0946

[48] Kaplan-Lefko PJ, Chen TM, Ittmann MM, Barrios RJ, Ayala GE, Huss WJ, et al Pathobiology of autochthonous prostate cancer in a pre-clinical transgenic mouse model. The Prostate. 2003;55(3):219–37. Epub 2003/04/15. 10.1002/pros.10215 . 12692788

[49] Slusarz A, Jackson GA, Day JK, Shenouda NS, Bogener JL, Browning JD, et al Aggressive prostate cancer is prevented in ERalphaKO mice and stimulated in ERbetaKO TRAMP mice. Endocrinology. 2012;153(9):4160–70. Epub 2012/07/04. 10.1210/en.2012-1030 22753646 PMC3423626

[50] Slusarz A, Shenouda NS, Sakla MS, Drenkhahn SK, Narula AS, MacDonald RS, et al Common botanical compounds inhibit the hedgehog signaling pathway in prostate cancer. Cancer Res. 2010;70(8):3382–90. Epub 2010/04/17. 10.1158/0008-5472.CAN-09-3012 . 20395211

[51] Sutton A, Buencamino R, Eisenstark A. rpoS mutants in archival cultures of Salmonella enterica serovar typhimurium. J Bacteriol. 2000;182(16):4375–9. . 10913067 10.1128/jb.182.16.4375-4379.2000 PMC94605

[52] Howlader N NA, Krapcho M, Garshell J, Miller D, Altekruse SF, Kosary CL, Yu M, Ruhl J, Tatalovich Z, Mariotto A, Lewis DR, Chen HS, Feuer EJ, Cronin KA (eds). SEER Cancer Statistics Review, 1975–2012. Bethesda, MD: National Cancer Institute, Based on November 2014 SEER data submission, posted to the SEER web site, April 2015.

[53] Harris WP, Mostaghel EA, Nelson PS, Montgomery B. Androgen deprivation therapy: progress in understanding mechanisms of resistance and optimizing androgen depletion. Nat Clin Pract Urol. 2009;6(2):76–85. 10.1038/ncpuro1296 19198621 PMC2981403

[54] Katzenwadel A, Wolf P. Androgen deprivation of prostate cancer: Leading to a therapeutic dead end. Cancer letters. 2015;367(1):12–7. 10.1016/j.canlet.2015.06.021 . 26185001

[55] Hirano D, Okada Y, Minei S, Takimoto Y, Nemoto N. Neuroendocrine differentiation in hormone refractory prostate cancer following androgen deprivation therapy. Eur Urol. 2004;45(5):586–92; discussion 92. 10.1016/j.eururo.2003.11.032 . 15082200

[56] Chang WW, Kuan YD, Chen MC, Lin ST, Lee CH. Tracking of mouse breast cancer stem-like cells with Salmonella. Exp Biol Med (Maywood). 2012;237(10):1189–96. 10.1258/ebm.2012.012063 . 23045719

[57] Hiroshima Y, Zhao M, Zhang Y, Maawy A, Hassanein MK, Uehara F, et al Comparison of efficacy of Salmonella typhimurium A1-R and chemotherapy on stem-like and non-stem human pancreatic cancer cells. Cell cycle (Georgetown, Tex. 2013;12(17):2774–80. 10.4161/cc.25872 23966167 PMC3899191

[58] Kasinskas RW, Forbes NS. Salmonella typhimurium specifically chemotax and proliferate in heterogeneous tumor tissue in vitro. Biotechnol Bioeng. 2006;94(4):710–21. Epub 2006/02/14. 10.1002/bit.20883 . 16470601

[59] Friedlos F, Lehouritis P, Ogilvie L, Hedley D, Davies L, Bermudes D, et al Attenuated Salmonella targets prodrug activating enzyme carboxypeptidase G2 to mouse melanoma and human breast and colon carcinomas for effective suicide gene therapy. Clin Cancer Res. 2008;14(13):4259–66. Epub 2008/07/03. 10.1158/1078-0432.CCR-07-4800 . 18594008

[60] Chen G, Tang B, Yang BY, Chen JX, Zhou JH, Li JH, et al Tumor-targeting Salmonella typhimurium, a natural tool for activation of prodrug 6MePdR and their combination therapy in murine melanoma model. Applied microbiology and biotechnology. 2012 Epub 2012/08/08. 10.1007/s00253-012-4321-8 . 22868826

[61] Ruoslahti E, Bhatia SN, Sailor MJ. Targeting of drugs and nanoparticles to tumors. The Journal of cell biology. 2010;188(6):759–68. Epub 2010/03/17. 10.1083/jcb.200910104 20231381 PMC2845077

[62] Avogadri F, Mittal D, Saccheri F, Sarrafiore M, Ciocca M, Larghi P, et al Intra-tumoral Salmonella typhimurium induces a systemic anti-tumor immune response that is directed by low-dose radiation to treat distal disease. European journal of immunology. 2008;38(7):1937–47. 10.1002/eji.200738035 . 18581324

[63] Kazmierczak R, Choe E, Sinclair J, Eisenstark A. Direct attachment of nanoparticle cargo to salmonella typhimurium membranes designed for combination bacteriotherapy against tumors. Methods in molecular biology (Clifton, NJ. 2015;1225:151–63. 10.1007/978-1-4939-1625-2_11 . 25253255

[64] Schatten H, Wiedemeier AM, Taylor M, Lubahn DB, Greenberg NM, Besch-Williford C, et al Centrosome-centriole abnormalities are markers for abnormal cell divisions and cancer in the transgenic adenocarcinoma mouse prostate (TRAMP) model. Biology of the cell / under the auspices of the European Cell Biology Organization. 2000;92(5):331–40. . 11071042 10.1016/s0248-4900(00)01079-0

[65] Bernardes N, Chakrabarty AM, Fialho AM. Engineering of bacterial strains and their products for cancer therapy. Applied microbiology and biotechnology. 2013;97(12):5189–99. Epub 2013/05/07. 10.1007/s00253-013-4926-6 . 23644748

[66] Sun Y, Niu J, Huang J. Neuroendocrine differentiation in prostate cancer. American journal of translational research. 2009;1(2):148–62. 19956427 PMC2776313

[67] Ittmann M, Huang J, Radaelli E, Martin P, Signoretti S, Sullivan R, et al Animal models of human prostate cancer: the consensus report of the New York meeting of the Mouse Models of Human Cancers Consortium Prostate Pathology Committee. Cancer Res. 2013;73(9):2718–36. 10.1158/0008-5472.CAN-12-4213 23610450 PMC3644021

[68] Qi J, Nakayama K, Cardiff RD, Borowsky AD, Kaul K, Williams R, et al Siah2-dependent concerted activity of HIF and FoxA2 regulates formation of neuroendocrine phenotype and neuroendocrine prostate tumors. Cancer cell. 2010;18(1):23–38. 10.1016/j.ccr.2010.05.024 20609350 PMC2919332

