# Supplementary material for: Salmonella Bacterial Monotherapy Reduces Autochthonous Prostate Tumor Burden in the TRAMP Mouse Model
Source: PLoS One. 2016 Aug 9;11(8):e0160926. doi: 10.1371/journal.pone.0160926 (PMC4978392; doi:10.1371/journal.pone.0160926)
Supplement: S1 Fig — The top graph shows the survival curves for all four groups with 95% confidence intervals. The bottom table is the product-limit survival estimates for the four groups without 95% confidence intervals. (PDF) [file pone.0160926.s001.pdf]

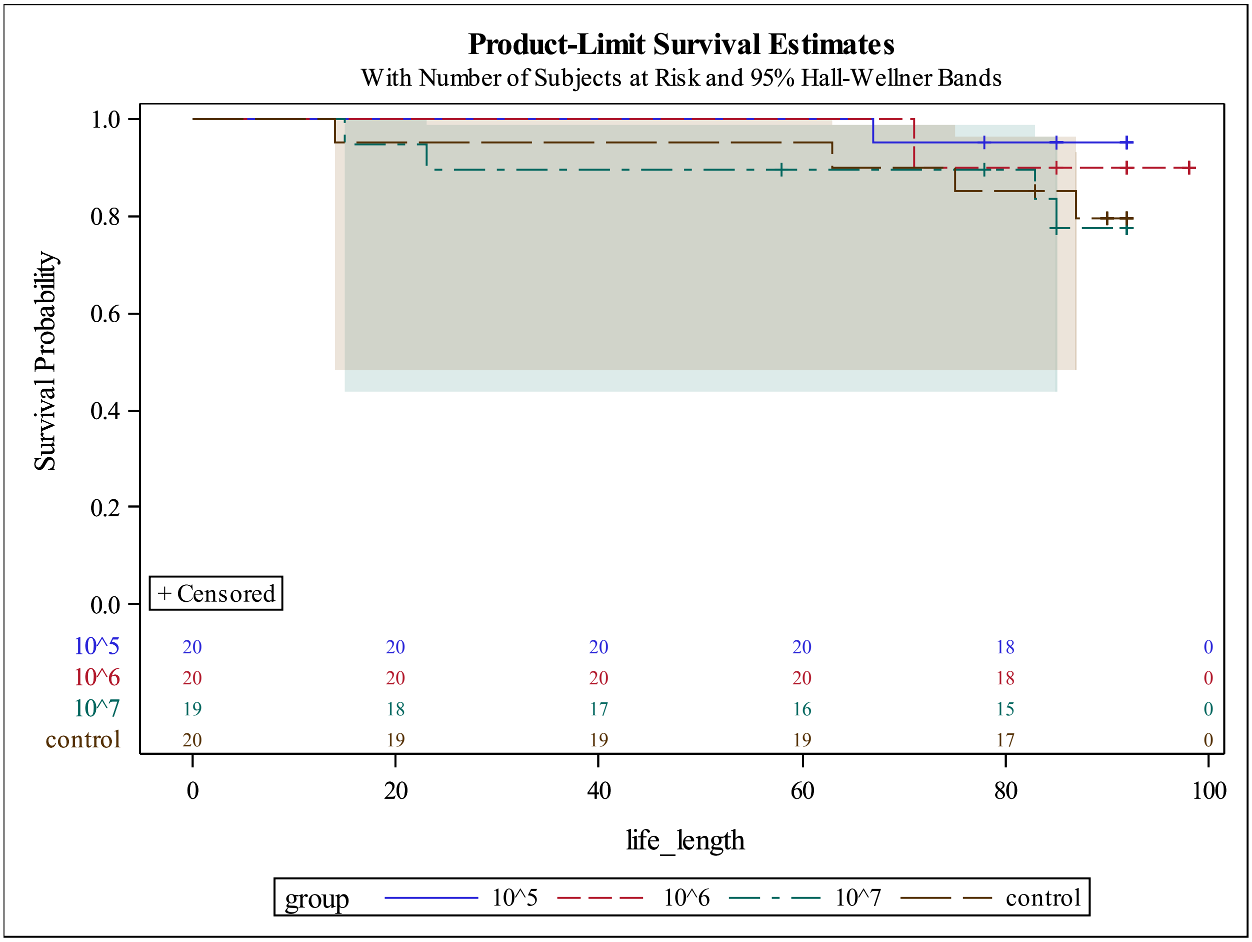

Test of Equality over Strata			
Test	Chi-Square	DF	Pr > Chi-Square
**Log-Rank**	2.9773	3	0.3951
**Wilcoxon**	2.8253	3	0.4194
**-2Log(LR)**	3.2267	3	0.3580

S1 Fig. Statistical Survival Curve Analysis. The top graph shows the survival curves for all four groups with 95% confidence intervals. The bottom table is the product-limit survival estimates for the four groups without 95% confidence intervals.
