# Supplementary material for: Salmonella Bacterial Monotherapy Reduces Autochthonous Prostate Tumor Burden in the TRAMP Mouse Model
Source: PLoS One. 2016 Aug 9;11(8):e0160926. doi: 10.1371/journal.pone.0160926 (PMC4978392; doi:10.1371/journal.pone.0160926)
Supplement: S1 File — TRAMP mice that scored 1.0 or greater on the pain/distress evaluation were euthanized after consultation with the attending veterinarian. (PDF) [file pone.0160926.s002.pdf]

Pain/Distress Evaluation for Rodents

PI________________________________ Protocol #_________
Animal ID_______________ Species________ Procedure______________
Date of Procedure__________Animal location_________

DATE										
DAY	1	2	3	4	5	6	7	8	9	10
TIME										
1. Attitude										
2. Harderian gland secretion										
3. Gait and Posture										
Bodyweight										
4. Weight Score										
5. Appetite										
6. Total Score										
If total score is ≥ 1.0 contact veterinarian or euthanize										
Other										
Signature										

Notes____

Attitude
0.0 Bright, alert, responsive
0.1 Burrowing or hiding, quiet but rouses when touched
0.4 No cage exploration when lid off, burrows/hides, may vocalize or be unusually aggressive when touched.

Harderian gland secretion
0.0 None
0.1 Mild around eyes and/or nostrils
0.4 Obvious on face and/or paws

Gait and Posture
0.0 Normal
0.2 Mild incoordination when stimulated, hunched posture, mild piloerection
0.4 Obvious ataxia or head tilt, hunching, drags one or both limbs, severe piloerection

Weight
0.0 up to 5% weight loss over pre-op weight days 1-3
0.2 5-10% weight loss over pre-op weight
0.4 10-20% weight loss over pre-op weight

Appetite
0.0 Normal, eats dry food, evidence of urine and feces, food missing from feeder or floor
0.1 No evidence of eating food but appears hydrated (skin does not "tent")
0.4 No interest in food and/or appears dehydrated (skin "tents")

S1 File. Pain/distress evaluation for rodents. TRAMP mice that scored 1.0 or greater on the pain/distress evaluation were euthanized after consultation with the attending veterinarian.
