# Supplementary material for: Salmonella Bacterial Monotherapy Reduces Autochthonous Prostate Tumor Burden in the TRAMP Mouse Model
Source: PLoS One. 2016 Aug 9;11(8):e0160926. doi: 10.1371/journal.pone.0160926 (PMC4978392; doi:10.1371/journal.pone.0160926)
Supplement: S1 Table — The Type 3 Tests for differences between the four groups gives a Wald Chi-Square of 2.6188 with 3 degrees of freedom (DF) and corresponds with a p-value of 0.4542. This tells us that there is not sufficient evidence to conclude that there are any differences in survival among the four groups. Further, we can look at the Analysis of Maximum Likelihood Estimates to see the Hazard Ratio (compared to the control group) and the p-values for comparison to the control group. All p-values are greater than 0.05 so we conclude that there is not sufficient evidence to conclude that any of the groups (10^5, 10^6, 10^7) have significantly different survival than the control group. (PDF) [file pone.0160926.s003.pdf]

Summary of the Number of Event and Censored Values			
Total	Event	Censored	Percent Censored
79	11	68	86.08

Testing Global Null Hypothesis: BETA=0			
Test	Chi-Square	DF	Pr > ChiSq
Likelihood Ratio	3.1623	3	0.3673
Score	2.9698	3	0.3963
Wald	2.6188	3	0.4542

Type 3 Tests			
Effect	DF	Wald Chi-Square	Pr > ChiSq
group	3	2.6188	0.4542

Analysis of Maximum Likelihood Estimates								
Parameter		DF	Parameter Estimate	Standard Error	Chi-Square	Pr > ChiSq	Hazard Ratio	Label
group	10^5	1	-1.38068	1.11975	1.5204	0.2176	0.251	group 10^5
group	10^6	1	-0.75785	0.86611	0.7656	0.3816	0.469	group 10^6
group	10^7	1	0.13408	0.70799	0.0359	0.8498	1.143	group 10^7

S1 Table. Statistical analysis of survival data between Salmonella injection groups. The Type 3 Tests for differences between the four groups gives a Wald Chi-Square of 2.6188 with 3 degrees of freedom (DF) and corresponds with a p-value of 0.4542. This tells us that there is not sufficient evidence to conclude that there are any differences in survival among the four groups. Further, we can look at the Analysis of Maximum Likelihood Estimates to see the Hazard Ratio (compared to the control group) and the p-values for comparison to the control group. All p-values are greater than 0.05 so we conclude that there is not sufficient evidence to conclude that any of the groups (10^5, 10^6, 10^7) have significantly different survival than the control group.
